# Differential miRNA expression profiles in variants of papillary thyroid carcinoma and encapsulated follicular thyroid tumours

**DOI:** 10.1038/sj.bjc.6605493

**Published:** 2009-12-22

**Authors:** S-Y Sheu, F Grabellus, S Schwertheim, K Worm, M Broecker-Preuss, K W Schmid

**Affiliations:** 1 Institute of Pathology and Neuropathology, University Hospital of Essen, University of Duisburg-Essen, Essen, Germany; 2 Department of Endocrinology and Division of Laboratory Research, University Hospital of Essen, University of Duisburg-Essen, Essen, Germany

**Keywords:** miRNA, papillary thyroid carcinoma, follicular variant, follicular adenoma, encapsulated follicular thyroid tumour, follicular thyroid carcinoma

## Abstract

**Background:**

Recent studies showed a significant upregulation of distinct microRNAs (miRNAs) in papillary thyroid carcinoma (PTC). The objective of this study was to explore whether this upregulation could also be assigned to distinct histomorphological variants of PTC, especially the follicular variant and other encapsulated follicular thyroid tumours.

**Methods:**

We used total RNA of 113 formalin-fixed paraffin-embedded tissues of 50 PTCs ((10 conventional type (PTC-CT), 10 tall cell variants (PTC-TCVs), 30 follicular variants (PTC-FVs)), 10 follicular adenomas (FAs), 10 multinodular goitres (MNGs), 21 follicular thyroid carcinomas and 22 well-differentiated tumours of unknown malignant potential (WDT-UMP) to analyse the miRNA expression pattern of five selected miRNAs (146b, 181b, 21, 221 and 222) using RT–PCR TaqMan miRNA assay to explore the diagnostic utility of this method.

**Results:**

The mean values of the expression pattern of all miRNAS in PTCs show a statistically significant difference from those in MNG and FA with fold changes up to 90 for miRNA 146b, *P*<0.001. No differences in expression pattern could be showed between MNG and FA. The PTC-FVs differ significantly from FA in all five miRNAS, from MNG in three and from WDT-UMP in one miRNA with fold changes between 1.7 and 21.2, but failed to be of diagnostic value regarding individual cases with substantial overlaps.

**Conclusion:**

We conclude that analysis of a set of five selected miRNAS distinguish common variants of PTC from FA/MNG but failed to be a useful diagnostic method in individual and doubtful cases, especially in the differential diagnosis of encapsulated follicular thyroid tumours.

Papillary thyroid carcinomas (PTCs) represent the most common thyroid malignancy; its diagnosis is based on the demonstration of characteristic nuclear features such as enlargement, overlapping, irregularity of nuclear contours, ground glass nuclei, grooves and pseudoinclusions (Rosai *et al*, 1992; DeLellis and Williams, 2004). However, PTCs comprise a morphologically heterogeneous group covering distinct variants that are classified on the basis of the occurrence of predominantly papillary structures (conventional type (PTC-CT)), a distinct growth pattern (follicular variant (PTC-FV) or cell type (e.g., tall cell variant (PTC-TCV)), among other features (DeLellis and Williams, 2004; LiVolsi and Baloch, 2004). This heterogeneity is also reflected in variable prevalences of the three most common genetic alterations, RET/PTC rearrangements (Nikiforov *et al*, 1997; Adeniran *et al*, 2006), *BRAF* (Trovisco *et al*, 2004; Xing, 2005) and *RAS* mutations (Vasko *et al*, 2003; Castro *et al*, 2006) that can be shown in approximately 70% of all PTCs (Nikiforova *et al*, 2008).

Since its original description by Crile and Hazard (1953) in 1953 and confirmation by Lindsay in 1960 (Lindsay, 1960) the PTC-FV represents a diagnostic challenge (Lloyd *et al*, 2004). Differential diagnostic problems are caused by the encapsulated form of PTC-FV that essentially has to be distinguished from other encapsulated lesions. With regard to therapeutic consequences, it is more important to differ PTC-FV from follicular adenoma (FA) than PTC-FV from minimally invasive follicular thyroid carcinoma (FTC). Moreover, pathologists are frequently faced with encapsulated thyroid tumours having ‘questionable’ PTC-type nuclear changes, as it has been pointed out by Rosai, (2005). Those tumours are referred to as ‘well-differentiated tumours of uncertain malignant potential’ (WDT-UMP) in the literature.

MicroRNAs (miRNAs) are endogenous, non-coding, small RNAs that regulate gene expression. A large number of miRNAs are involved in almost every major cellular function (Cowland *et al*, 2007) and as a consequence, deregulation of miRNAs has also been linked to a broad variety of cancers (Calin *et al*, 2002, 2005; Michael *et al*, 2003; Takamizawa *et al*, 2004; Iorio *et al*, 2005; Lu *et al*, 2005; Mattie *et al*, 2006; Murakami *et al*, 2006; Volinia *et al*, 2006). Recently, a few studies reported on deregulated miRNAs in PTC using miRNA microarrays (He *et al*, 2005; Pallante *et al*, 2006; Tetzlaff *et al*, 2007; Nikiforova *et al*, 2008) and RT–PCR TaqMan miRNA assay (Tetzlaff *et al*, 2007; Chen *et al*, 2008; Nikiforova *et al*, 2008), identifying a limited number of miRNAs that are significantly upregulated in PTC compared with normal thyroid tissue (He *et al*, 2005; Pallante *et al*, 2006; Chen *et al*, 2008; Nikiforova *et al*, 2008), hyperplastic nodules (Chen *et al*, 2008; Nikiforova *et al*, 2008) and multinodular goitre (Tetzlaff *et al*, 2007; for review, see Table 1), suggesting miRNA analysis as a promising tool in diagnostic thyroid pathology.

With regard to both the morphologic and genetic differences between PTC variants, we asked whether analysis of a distinct set of miRNAs is able to reliably distinguish common variants of PTC (PTC-CT, PTC-TCV and PTC-FV) from multinodular goitre (MNG) and FA and whether miRNA expression profiling is a useful tool in the differential diagnosis of encapsulated follicular thyroid tumours.

## Materials and methods

### Patients and tumour samples

For this study we selected the following cases from the files of the Institute of Pathology and Neuropathology, University Hospital of Essen, Germany: 10 cases with an (almost) exclusive papillary architecture, the characteristic nuclear features outlined by the WHO classification (2004), and particularly lacking both the cellular and nuclear features of the tall cell variant of PTC that belong to the conventional-type group (PTC-CT). Another 10 cases were categorised as the tall cell variant of PTC (PTC-TCV). These tumours showed tumour cells at least twice high than wide with an abundant eosinophilic cytoplasm and typical nuclear characteristics, including eosinophilic pseudoinclusions. To explore differences in miRNA expression, especially in encapsulated follicular thyroid tumours, we selected 30 cases composed of >95% of follicular structures and characteristic nuclear features corresponding to the follicular variant of PTC (PTC-FV). Out of 50 PTCs, seven had been previously analysed for miRNA analysis (Schwertheim *et al*, 2009; Sheu *et al*, 2009). A total of 21 minimally invasive FTCs with either capsular (*n*=6) or vascular invasion (*n*=13) or both (*n*=2) were also included. In addition, we selected 22 encapsulated follicular tumours with questionable nuclear changes without vascular/capsular invasion; these tumours are categorised as WDT-UMP. A total of 10 encapsulated thyroid FAs and 10 MNGs were also included. All patients gave informed consent and the study was strictly performed according to the Declaration of Helsinki.

### Macrodissection of tumour tissue

Macrodissection from paraffin-embedded specimens to obtain ‘pure’ tumour tissues was performed as described before (Sheu *et al*, 2007). From all cases, at least three tissue blocks were available and ‘morphologic homogeneity’, especially in variants of PTC, was proven in all blocks. Clinicopathologic data of all cases are summarised in Table 2.

### RNA extraction

RNA was extracted using the RNeasy FFPE Kit (Qiagen, Hilden, Germany). In brief, tissue sections were deparaffinised by xylene/ethanol treatment. Tissue pellets were resuspended in 150 *μ*l buffer PKD, 20 *μ*l proteinase K and incubated overnight on a shaker incubator at 56 °C. Further processing of the samples was performed according to the recommendations of the supplier.

### Selection and detection of miRNAs

For this study we selected a set of five miRNAs (miRNAs 146b, 181b, 21, 221 and 222) that are significantly upregulated in PTC compared with normal thyroid tissue (He *et al*, 2005; Pallante *et al*, 2006; Nikiforova *et al*, 2008), hyperplastic nodules (Nikiforova *et al*, 2008) and multinodular goiter (Tetzlaff *et al*, 2007). This set of miRNAs was analysed using the real-time RT–PCR scheme for miRNA quantification according to the protocol of Applied Biosystems (P/N: 4364031); this two-step protocol consists of reverse transcription with a miRNA-specific primer, followed by real-time PCR with TaqMan probes. The TaqMan miRNA assays used were also provided by Applied Biosystems. In brief, for each RT–PCR 50 ng RNA was reverse transcripted to cDNA using 3 *μ*l specific looped RT primers (Applied Biosystems, Darmstadt, Germany and 200 U MuLV reverse transcriptase (Fermantas, Vilnius, Lithuania). The 15 *μ*l reactions were incubated in a Primus 25 thermocycler (MWG Biotech, Ebersberg, Germany) for 30 min at 16 °C, 30 min at 42 °C, 5 min at 85 °C and then kept at 4 °C. Real-time PCR was performed in triplicate using a standard protocol on the Applied Biosystems 7500 Sequence Detection System. Each PCR included 5.25 *μ*l of a 1 : 25 dilution of specific cDNA in water, 1 *μ*l of the specific miRNA Assay Mix and 6.25 *μ*l of 2 × Taq Man Universal PCR Master Mix. The reactions were incubated in a 96-well plate at 95 °C for 10 min, followed by 40 cycles at 95 °C for 15 s and 60 °C for 1 min. In each sample the relative amount of miRNA was calculated using the comparative threshold method determining RNU 48 as the endogenous control with ΔCt=Ct (miRNA) – Ct (RNU48). Relative quantification of miRNA expression was calculated with the 2^−ΔΔCt^ method (Applied Biosystems user bulletin no. 2 (P/N 4303859)). This method facilitates detecting and quantifies exclusively mature miRNAs but not their precursors.

### Statistical analysis

Statistical analysis was performed using the Statistical package for Social Sciences (SPSS; Version 17.0 for Windows, Chicago, IL, USA). Correlations between different mean ΔCt values and relative quantification of miRNA expression were assessed using the Mann–Whitney test for two unpaired groups.

## Results

### miRNA expression patterns in all PTC *vs* benign thyroid lesions (FA and MNG)

Comparison of mean ΔCt values of PTC as a group, including all variants, and FA and MNG, respectively, show a statistically significant difference in the expression pattern of all miRNAs (*P*⩽0.012) with lower mean values in all PTC samples analysed (Table 3). Calculating relative changes in gene expression by the 2^−ΔΔCt^ method (Livak and Schmittgen, 2001) showed a 80- to 90-fold change of miRNA 146b in all PTC cases (*P*<0.001; Table 3) for both groups (PTC *vs* MNG and PTC *vs* FA). Fold changes varied between 0.8 and 16.4 for the other miRNAs tested. All five miRNAs that were analysed lacked significant differences between MNG and FA.

### miRNA expression pattern in variants of PTC

**Figure 1:**
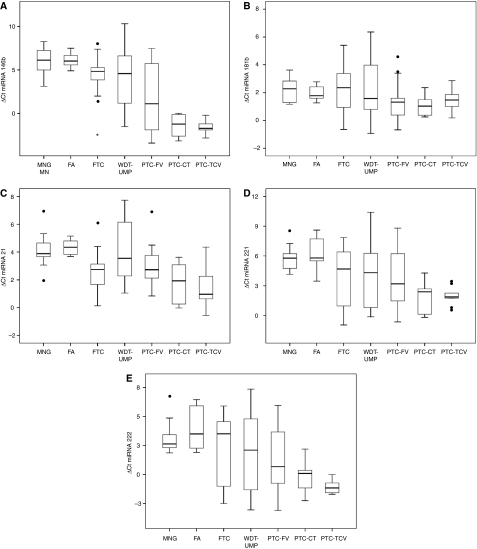
Different miRNA expression profiles of PTC variants, encapsulated benign and malignant lesions. Only miRNA 146b distinguishes every single PTC-TCV and PTC-CT from MNG and FA (**A**) whereas miRNAs 221 (**D**) and 222 (**E**) showed remarkable differences as well but failed to be a reliable diagnostic tool. PTC-FV and WDT-UMP revealed a broad variability in every type of miRNA analysed, covering the whole miRNA expression range that do not allow a clear distinction among tumour types. Mean values of miRNAs 146b and 21 differ significantly between FA and FTC in miRNA 21 (*P*=0.004; *P*<0.001).

The miRNA patterns of different variants of PTC are depicted in Figure 1. Whereas PTC-CT and PTC-TCV did not differ significantly in mean ΔCt values and consecutively fold changes of gene expression for all types of miRNA, the miRNA patterns of both PTC-CT and PTC-TCV differed from PTC-FV. When comparing mean values of PTC-CT and PTC-FV, a significant difference was exclusively found for miRNA 146b (fold change 8.0; *P*=0.043). Mean ΔCt values of PTC-TCV differed significantly from PTC-FV in three miRNAs (146b, 21 and 222; *P*<0.028) that correspond to fold changes between 2.9 and 9.9.

Regarding individual cases of PTC-FV, we observed a broad variability in every miRNA analysed. This variability covered the whole range of ΔCt values of benign (MNG and FA) and also TCV and CT of PTC. However, there was no overlap between every single case of PTC-CT and PTC-TCV and MNG/FA, showing that 146b is the only miRNA to reliably discriminate between PTC (conventional and tall cell variant) and benign thyroid lesions. Minimal overlaps exist concerning miRNA 221 and 222 in single cases that do not qualify these types to reliably distinguish between these two variants of PTC and MNG/FA.

### miRNA expression pattern in encapsulated follicular tumours

Taking out PTC-FV as a group, we looked for differences in expression pattern compared with benign thyroid lesions (Fig. 1 and Table 4). As it could already be observed for all PTCs (Table 3) the follicular variant also showed significant differences in mean ΔCt values in at least three of five examined miRNA types compared with FA and MNG. Only miRNA 146b was upregulated when comparing mean values of PTC-FV with WDT-UMP and FTC. Surprisingly, mean values of miRNA 146b and 21 differed in FA compared with FTC (*P*⩽0.004), whereas only miRNA 21 showed a fold change of 3.0 between WDT-UMP and FTC (*P*=0.027). Regarding miRNA deregulation in individual cases of PTC-FV there was also a broad overlap within all analysed encapsulated thyroid tumours whether they showed partial nuclear features of PTC (WDT-UMP) or not (FTC).

## Discussion

In accordance with previous studies (He *et al*, 2005; Pallante *et al*, 2006; Tetzlaff *et al*, 2007; Chen *et al*, 2008; Nikiforova *et al*, 2008) the analysis of a limited set of miRNAs represents a reliable method to distinguish PTC from benign thyroid lesions (for review see Table 1). Considering the mean ΔCt values of all miRNAs analysed in our study, we showed highly significant changes between PTC and MNG/FA. However, the most common variants of PTC show a different miRNA expression pattern with similar profiles of PTC-CT and PTC-TCV in contrast to the follicular variant of PTC. As for diagnostic purposes, only miRNA 146b reliably distinguish the conventional and tall cell variant from benign thyroid lesions in individual cases, whereas all other miRNAs show substantial overlap. This is in accordance with a study by Chen *et al* (2008) who found miRNA 146b to be most consistently overexpressed in both conventional and follicular variants when compared with ‘borderline’ follicular lesions, although the number of five follicular variants in their study is rather small.

The functional relevance of overexpressed miRNA 146b and its effect on PTC tumourigenesis had been elucidated in two studies (Taganov *et al*, 2006; Jazdzewski *et al*, 2008) so far. Taganov *et al*, (2006) identified TNF receptor-associated factor 6 (TRAF6) and IL-1 receptor-associated kinase (IRAK1), which represent potential molecular targets of miRNA 146, as modulating the immune response in a NF-*κ*B-dependent manner. As far as NF-*κ*B is one of the key factors controlling anti-apoptotic response in thyroid cells, it is also modulated by activated MAPK (Namba *et al*, 2007) in PTC. This pathway, in turn, is involved in downstream effects of RET/PTC rearrangements, *RAS* and *BRAF* mutations, the latter being most frequently verifiable in PTC. Therefore, it seems not surprising that upregulation of miRNA 146 is more distinctive in the conventional and tall cell variant of PTC, as these variants are significantly associated with the common V600E *BRAF* mutation. However, in a previous study we asked for a possible correlation between the occurrence of V600E *BRAF* mutation and miRNA expression profile and found no significant differences in miRNA expression between PTC harbouring the *BRAF* mutation and wild-type *BRAF,* implicating that this mutation has no *regulatory* influence on the expression pattern of these 5 miRNAs (Sheu *et al*, 2009). Our results are in contrast to another study by Nikiforova *et al* (2008) who founded a strong relationship between miRNA expression and mutational status (*BRAF*, *RET/PTC* and *PAX8-PPARγ*). The reported differences might be due to the variable number of tissue samples (28 *vs* 6) harbouring V600E mutation and probably different statistical analysis and illustration (raw data presenting ΔCt values *vs* principal component analysis (PCA)). This possible relationship should be validated in a larger cohort of PTCs.

The miRNA pattern of PTC-FV in this study differed from PTC-TCV in three (miRNAs 146b, 21 and 222) and from PTC-CT in 1 miRNA (miRNA 146b). In our previous study (Sheu *et al*, 2009) we normalised fold changes in PTC variants to adjacent normal thyroid tissue in a pairwise manner and found that follicular variants showed 3/5 upregulated miRNAs (146b, 221 and 222), whereas the conventional type differed in 4/5 (146b, 181b, 221, and 222) and tall cells in all examined miRNAs, indicating a heterogeneous regulatory role of certain miRNAs within PTCs. Although PTCs as a group of tumours sharing cytologic similarities showed a distinct upregulated miRNA pattern, differences in various genetic alterations among PTC variants are quite common. The V600E *BRAF* mutation has been shown in approximately 43% of PTC (Lupi *et al*, 2007), ranging from 12% in PTC-FV to 77% in PTC-TCV (Xing, 2005). Interestingly, a distinct *BRAF* mutation (K601E) had been exclusively found in PTC-FV (Trovisco *et al*, 2004, 2005) and in FA (Lima *et al*, 2004). The genetic and morphologic overlap of PTC-FV is also supported by the results of Castro *et al* (2006) who found similar frequencies of activating point mutations of the *RAS* genes and *PAX8-PPARγ* rearrangement in PTC-FV, FA and FTC; both genetic alterations are absent (*PAX8-PPARγ* Kroll *et al*, 2000; Nikiforova *et al*, 2002) or exceedingly rarely (*RAS*; Vasko *et al*, 2003) found in ‘non follicular variant’ of PTC.

However, the comparison of miRNA expression profile of PTC-FV and other encapsulated follicular thyroid lesions revealed a broad variability among individual cases with substantial overlap. We observed a similar broad ΔCt range especially in PTC-FV and WDT-UMPs, reflecting that both tumours not only share distinct morphologic characteristics but also similarities in miRNA regulation. As for practical purposes, the determination of miRNA expression profile of our types analysed does not contribute to clarify the biological significance of those tumours assuming that there might be other factors than the characteristic nuclear features in the ‘majority of the tumour’ of PTC-FV as outlined in the WHO classification. Regarding clinical and therapeutical consequences, especially the discrimination between FA and PTC-FV and FA *vs* FTC, respectively, the miRNA expression analysis also failed to be of diagnostic value, although highly significant mean ΔCt values and consecutively fold changes up to approximately 21 (for 146b) between PTC-FV and FA indicate remarkable differences. However, these changes could only be showed in mean values but not in individual cases. Surprisingly and in addition to the identified miRNAs (197, 328, 346 and 192) by Weber (Weber *et al*, 2006) we found differences in miRNA expression pattern between FA and FTC with fold changes between 3.1 and 3.5 for miRNAs 146b and 21, and in accordance with their study, not for miRNAs 221 and 221, indicating the latter two being essentially involved in PTC pathogenesis, as it has previously been shown by Pallante (Pallante *et al*, 2006). This points towards the role of miRNA 146b as being generally involved in both (papillary and follicular) phenotypes of thyroid carcinogenesis, reflecting other genetic alterations that partly result in characteristic nuclear features. In our study, and as for the distinction between FA and FTC, miRNA 21 seem to have a regulatory role, as an upregulation of miRNA could recently be shown in *RAS-*transformed FRTL-5 thyroid cells (Talotta *et al*, 2009). Two targets of miRNA 21, the tumour suppressor genes PTEN and PDCD4, are downregulated in a novel autoregulatory loop mediated by miRNA 21 through the transcription factor AP1 in response to *RAS*, thus indicating a tumourigenetic role for miRNA, but they failed to be of diagnostic value in every single case in our study.

We have shown that an analysis of a set of five selected miRNAs distinguish common variants of PTC from follicular adenoma and multinodular goitre but failed to be a useful diagnostic method in individual and doubtful cases, especially in the differential diagnosis of follicular thyroid tumours (PTC-FV, FA, WDT-UMP and FTC). In addition, miRNA expression profiling confirms the so far ‘intermediate’ position of PTC-FV between conventional and tall cell variants of PTCs on one hand, and on the other hand, the follicular thyroid tumours with partly nuclear features of unknown malignant potential and minimal invasive FTC.

## Figures and Tables

**Table 1:** Literature review over miRNA quantitative RT–PCR studies in thyroid tumours

**Material, Total sample**	**Tumour type/normal thyroid**	**Deregulated miRNA**	**Reference**
Fresh-frozen tissue, *n*=10	Matched pairs of 5 PTCs and 5 normal thyroids	miRNAs 221, 222 and 146b upregulated in PTC *vs* normal thyroid	He *et al* (2005)
Snap-frozen tissue, *n*=9	5 FTCs 4, FAs 4, normal thyroids	miRNAs 197 and 56 upregulated in FTC *vs* FA	Weber *et al* (2006)
Fresh frozen tissue, *n*=47	8 FAs and 39 PTCs	Precursor miRNAs 221, 222 and 181b-1 upregulated in PTC *vs* FA	Pallante *et al* (2006)
Formalin-fixed paraffin-embedded tissue, *n*=20	10 PTCs (conventional variant) and 10 MNGs	miRNAs 21, 31, 221 and 222 upregulated in PTC *vs* MNG	Tetzlaff *et al* (2007)
Fresh-frozen and formalin-fixed paraffin-embedded tissue, *n*=20	20 ATCs	miRNAs 30d, 125b, 26 and 30-a-5p downregulated in ATC *vs* normal thyroid	Visone *et al* (2007)
Snap-frozen tissue, *n*=60	23 PTCs (18 conventional and 5 follicular variants) 9 FTCs, 8 FAs, 4 ACs, 4 PDCs, 2 MCs, 5 normal thyroids and 5 hyperplastic nodules	miRNAs 187, 221, 222, 181b, 146b, 155 and 224 upregulated in follicular cell-derived carcinomas with high variance between tumour type; miRNAs 187, 221, 222, 146b and 155 upregulated in PTC with strong relationship to mutational status	Nikiforova *et al* (2008)
Formalin-fixed paraffin-embedded tissue, *n*=74	32 PTCs (27 conventional and 5 follicular variants) 24 FAs, 10 ‘borderline’ follicular lesions, 11 hyperplastic nodules, 2 FTCs and 5 normal thyroid	miRNA 146 upregulated in PTC *vs* non-papillary carcinomas; miRNAs 221 and 222 upregulated in PTC *vs* FA, hyperplastic nodules, normal thyroid but with substantial overlaps in individual cases; miRNA 146b indistinguishable between ‘borderline’ tumours and FA and lower than in PTC	Chen *et al* (2008)
Formalin-fixed paraffin-embedded tissue, *n*=113	50 PTCs (10 conventional, 10 tall cell and 30 follicular variants) 22 WDT-UMPs, 21 FTCs 10 FAs and 10 MNGs	Mean values of miRNAs 146b, 181b, 21, 221 and 222 upregulated in PTC; miRNA 146b upregulated in PTC-CT/PTC-TCV *vs* MNG/FA; substantial overlaps of all miRNAs in follicular cell-derived tumours; intermediate miRNA expression profile of PTC-FV	Our study

Abbreviations: PTC=papillary thyroid carcinoma; FTC=follicular thyroid carcinoma; FA=follicular adenoma; MNG=multinodular goitre; ATC/AC=anaplastic carcinoma; PDC=poorly differentiated carcinoma; MC=medullary carcinoma; WDT-UMP=well-differentiated tumour of unknown malignant potential.

**Table 2:** Clinicopathological features of all cases

	**No. of cases**	**Male/female ratio**	**Mean age (year)±s.d.**	**Mean tumour size (mm)±s.d.**
All samples	113	1 : 2.8	46.6±15.2	27.3±14.8
Diagnosis				
MNG	10	1 : 4	49.5±10.1	
FA	10	1.5 : 1	46.7±10.3	25.4±11.5
				
*PTC*	50	1 : 2.8	49.2±15.2	27.3±17.1
Conventional	10	1 : 4	48.1±14.0	26.2±18.8
Tall cell	10	1 : 9	58.6±12.7	24.9±13.5
Follicular	30	1 : 2	46.4±15.4	28.5±18.1
				
*FTC with*	21	1 : 9.5	44.3±18.3	27.5±13.5
Vascular invasion	13			
Capsular invasion	6			
Both	2			
WDT-UMP	22	1 : 2.1	41.3±15.5	27.7±11.8

Abbreviations: MNG=multinodular goitre, FA=follicular adenoma, PTC=papillary thyroid carcinoma, FTC=follicular thyroid carcinoma, WDT-UMP=well-differentiated tumour of unknown malignant potential.

**Table 3:** Mean ΔCt values and fold changes in PTC, MNG and FA

			**PTC^a^ *vs* MNG**		**PTC^a^ *vs* FA**		**MNG *vs* FA**	
**miRNA type**	**Tumour tissue**	**Mean ΔCt**	**Fold change**	***P-*value**	**Fold change**	***P***-**value**	**Fold change**	***P*-value**
146b	MNG	5.922						
	FA	6.092	81.3	<0.001	91.4	<0.001	1.1	NS
	PTC^a^	−0.422						
181b	MNG	2.239						
	FA	1.986	2.1	0.012	1.7	0.003	0.8	NS
	PTC^a^	1.200						
21	MNG	4.159						
	FA	4.338	4.4	0.001	4.9	<0.001	1.1	NS
	PTC^a^	2.032						
221	MNG	5.835						
	FA	6.289	9.4	0.002	12.9	0.001	1.4	NS
	PTC^a^	2.605						
222	MNG	3.183						
	FA	3.992	9.4	0.001	16.4	<0.001	1.8	NS
	PTC^a^	−0.044						

a PTC includes all variants of PTC (conventional type, tall cell variant and follicular variant).

**Table 4:** Mean ΔCt values and fold changes in encapsulated follicular thyroid lesions

			**PTC-FV *vs* FA**		**PTC-FV *vs* MNG**		**PTC-FV *vs* WDT-UMP**		**PTC-FV *vs* FTC**		**FA *vs* FTC**		**WDT-UMP *vs* FTC**	
**miRNA type**	**Tumour tissue**	**Mean ΔCt**	**Fold change**	***P*-value**	**Fold change**	***P*-value**	**Fold change**	***P*-value**	**Fold change**	***P*-value**	**Fold change**	***P*-value**	**Fold change**	***P*-value**
146b	MNG	5.922												
	WDTUMP	4.148	21.2	0.001	18.9	0.002	5.5	0.002	6.8	0.042	3.1	0.004	0.8	NS
	PTC-FV	1.684												
	FA	6.092												
	FTC	4.447												
181b	MNG	2.239												
	WDTUMP	2.200	1.7	0.005	2.0	0.024	2.0	NS	2.0	NS	0.9	NS	1.0	NS
	PTC-FV	1.206												
	FA	1.986												
	FTC	2.218												
21	MNG	4.159												
	WDTUMP	4.095	2.6	<0.001	2.3	0.011	2.2	NS	0.7	NS	3.5	<0.001	3.0	0.027
	PTC-FV	2.935												
	FA	4.338												
	FTC	2.515												
221	MNG	5.835												
	WDTUMP	4.178	5.3	0.020	3.9	NS	1.2	NS	0.9	N.S.	5.6	NS	1.3	NS
	PTC-FV	3.887												
	FA	6.289												
	FTC	3.812												
222	MNG	3.183												
	WDTUMP	2.086	6.8	0.005	3.9	NS	1.8	NS	1.9	NS	3.5	NS	0.9	NS
	PTC-FV	1.237												
	FA	3.992												
	FTC	2.200												
